# Non-Contact Measurement of Blood Oxygen Saturation Using Facial Video Without Reference Values

**DOI:** 10.1109/JTEHM.2023.3318643

**Published:** 2023-09-25

**Authors:** Soma Sasaki, Norihiro Sugita, Takanori Terai, Makoto Yoshizawa

**Affiliations:** Graduate School of EngineeringTohoku University 13101 Sendai 9808579 Japan; Cyberscience CenterTohoku University 13101 Sendai 9808579 Japan; Center for Promotion of Innovation StrategyTohoku University 13101 Sendai 9800845 Japan

**Keywords:** Facial video image, multispectral camera, percutaneous oxygen saturation, principal component analysis, video plethysmography

## Abstract

The continuous measurement of percutaneous oxygen saturation (SpO2) enables diseases that cause hypoxemia to be detected early and patients’ conditions to be monitored. Currently, SpO2 is mainly measured using a pulse oximeter, which, owing to its simplicity, can be used in clinical settings and at home. However, the pulse oximeter requires a sensor to be in contact with the skin; therefore, prolonged use of the pulse oximeter for neonates or patients with sensitive skin may cause local inflammation or stress due to restricted movement. In addition, owing to COVID-19, there has been a growing demand for the contactless measurement of SpO2. Several studies on measuring SpO2 without contact used skin video images have been conducted. However, in these studies, the SpO2 values were estimated using a linear regression model or a look-up table that required reference values obtained using a contact-type pulse oximeter. In this study, we propose a new technique for the contactless measurement of SpO2 that does not require reference values. Specifically, we used certain approaches that reduced the influence of non-pulsating components and utilized different light wavelengths of video images that penetrated subcutaneously to different depths. We experimentally investigated the accuracy of SpO2 measurements using the proposed methods. The results indicate that the proposed methods were more accurate than the conventional method.

## I. Introduction

Information on the amount of oxygen contained in blood is important for evaluating the oxygen exchange function of the lungs. In particular, the saturation of arterial oxygen (SaO2) and percutaneous oxygen (SpO2) represent the percentage of hemoglobin bound to oxygen from the total hemoglobin in the arterial blood. These values can be used for the early detection of hypoxemia-related diseases, e.g., pneumonia and chronic obstructive pulmonary disease (COPD) [1], [2] and monitoring newborns and patients under anesthesia [3], [4]. SaO_2_ is measured by taking blood samples from the arteries at the elbow or wrist and testing the obtained arterial blood with a blood gas analyzer. However, the value of SpO_2_ can be obtained percutaneously and accurately reflects SaO_2_ [5], [6]. The value of SpO_2_ is defined as

\begin{equation*} \mathrm {Sp}\mathrm {O}_{2}\equiv \frac {c_{\mathrm {Hb}\mathrm {O}_{2}}}{c_{\mathrm {Hb}\mathrm {O}_{2}}+c_{\mathrm {Hb}}}\times 100 \left ({}\right) \tag{1}\end{equation*}

where $c_{\mathrm {Hb}\mathrm {O}_{2}}$ and $c_{\mathrm {Hb}}$ are the molar concentrations of oxyhemoglobin and deoxyhemoglobin, respectively. Generally, the SpO_2_ of healthy people at rest ranges from 96% to 98% [7]. The value of SpO_2_ can be obtained continuously with a pulse oximeter by attaching a probe to the fingertip or ear. Because of its simplicity, a pulse oximeter is used clinically and for health management during home care and mountain climbing [8].

However, a pulse oximeter requires the sensor to be in contact with the skin during the measurement. Therefore, long-term monitoring of SpO_2_ in newborns and hospitalized patients with fragile skin may cause skin inflammation or severe stress. Hence, there is a need for a method to measure SpO_2_ without skin contact. Furthermore, interest in the non-contact measurement of SpO2 has grown due to the COVID-19 pandemic.

In recent years, contactless measurements using video cameras have attracted increasing attention for applications in biological monitoring. Verkruysse et al. [9] showed that the heart rate can be estimated based on slight changes in the intensity of light reflected from the skin, as captured by a visible-light camera. Video plethysmography (VPG), which utilizes pulse waves obtained from such videos, can analyze subcutaneous blood flow and the state of blood vessels. It has been applied to measure the heart rate and a range of other vital signs such as blood pressure [10], [11], [12], [13]. Some studies proposed techniques to estimate SpO_2_ from VPG signals [14], [15], [16], [17], [18], [19]. However, in these studies, a linear regression equation or a look-up table derived from reference values was used to estimate SpO_2_; thus, measurements using a contact-type pulse oximeter were necessary to create an estimation model for each subject or subject group.

This study aimed to establish a non-contact measurement method for SpO_2_ without using reference values. We proposed two approaches to extract only the components related to changes in hemoglobin levels from VPG signals. The first approach attempted to extract the components by separating the pulsatile and non-pulsatile components based on a model of light transmission under the skin, while the second attempted to extract them by applying principal component analysis (PCA) to VPG signals measured with different light wavelengths.

## II. Methods

### A. Non-Contact Measurement of Blood Oxygen Saturation Using Video Images of the Skin

We assume that $I_{0}\left ({\lambda }\right)$ and $I\left ({\lambda }\right)$ respectively are the incident and reflected light intensities. When the skin is illuminated by light of wavelength $\lambda $, based on Lambert–Beer’s law, $I\left ({\lambda }\right)$ is expressed as follows [20]:

\begin{equation*} I\left ({\lambda }\right)=I_{0}\left ({\lambda }\right)\cdot \exp \left \{{ -A\left ({\lambda }\right) }\right \} \tag{2}\end{equation*}

Here, $A\left ({\lambda }\right)$ is the absorbance in the skin and is expressed as follows:

\begin{align*}&\hspace {-.5pc} A\left ({\lambda }\right)=A_{\mathrm {m}}\left ({\lambda }\right)+A_{0}\left ({\lambda }\right) \\&\qquad\qquad\qquad +\,\left \{{\varepsilon _{\mathrm {Hb}\mathrm {O}_{2}}\left ({\lambda }\right)c_{\mathrm {Hb}\mathrm {O}_{2}}+\varepsilon _{\mathrm {Hb}}\left ({\lambda }\right)c_{\mathrm {Hb}} }\right \}l \tag{3}\end{align*}

where $A_{\mathrm {m}}(\lambda)$ and $A_{0}(\lambda)$ are the absorption due to melanin in the epidermis and all substances in the dermis; $\varepsilon _{\mathrm {Hb}\mathrm {O}_{2}}\left ({\lambda }\right) $ and $\varepsilon _{\mathrm {Hb}}\left ({\lambda }\right)$ are the molar absorption coefficients, $c_{\mathrm {Hb}\mathrm {O}_{2}}$ and $c_{\mathrm {Hb}}$ are the molar concentration coefficients of oxyhemoglobin and deoxyhemoglobin. $l$ is the mean optical path length.

Now, suppose that heart pulsation changes the optical path length by $\mathrm {\Delta }l$, causing the skin’s absorbance to change by $\mathrm {\Delta }A\left ({\lambda }\right)$. Here, we assume that $\mathrm {\Delta }A\left ({\lambda }\right)$ is mainly caused by changes in the blood vessels and not by other factors such as melanin. $\mathrm {\Delta }A\left ({\lambda }\right)$ is obtained from (3) as follows:

\begin{equation*} \Delta A\left ({\lambda }\right)=\left \{{\varepsilon _{\mathrm {Hb}\mathrm {O}_{2}}\left ({\lambda }\right)c_{\mathrm {Hb}\mathrm {O}_{2}}+\varepsilon _{\mathrm {Hb}}\left ({\lambda }\right)c_{\mathrm {Hb}} }\right \}\mathrm {\Delta }l \tag{4}\end{equation*}

The ratio of the absorbance changes for light with two different wavelengths, $\lambda _{1}$ and $\lambda _{2}$, is expressed as follows:

\begin{equation*} \frac {\Delta A\left ({\lambda _{1} }\right)}{\Delta A\left ({\lambda _{2} }\right)}=\frac {\varepsilon _{\mathrm {Hb}\mathrm {O}_{2}}\left ({\lambda _{1} }\right)c_{\mathrm {Hb}\mathrm {O}_{2}}+\varepsilon _{\mathrm {Hb}}\left ({\lambda _{1} }\right)c_{\mathrm {Hb}}}{\varepsilon _{\mathrm {Hb}\mathrm {O}_{2}}\left ({\lambda _{2} }\right)c_{\mathrm {Hb}\mathrm {O}_{2}}+\varepsilon _{\mathrm {Hb}}\left ({\lambda _{2} }\right)c_{\mathrm {Hb}}} \tag{5}\end{equation*}

Finally, the value of SpO_2_ can be obtained using (1) and (5):

\begin{align*}&\hspace {-.5pc} \mathrm {Sp}\mathrm {O}_{2} \\&= \frac {\varepsilon _{\mathrm {Hb}}\left ({\lambda _{1} }\right)-\varepsilon _{\mathrm {Hb}}\left ({\lambda _{2} }\right)\frac {\Delta A\left ({\lambda _{1} }\right)}{\mathrm {\Delta }A\left ({\lambda _{2} }\right)}}{\varepsilon _{\mathrm {Hb}}\left ({\lambda _{1} }\right)\!-\!\varepsilon _{{\mathrm {HbO}}_{2}}\left ({\lambda _{1} }\right)\!+\!\left \{{\varepsilon _{{\mathrm {HbO}}_{2}}\left ({\lambda _{2} }\right)\!-\!\varepsilon _{\mathrm {Hb}}\left ({\lambda _{2} }\right) }\right \}\frac {\Delta A\left ({\lambda _{1} }\right)}{\mathrm {\Delta }A\left ({\lambda _{2} }\right)}} \\&\quad \times \, 100 \tag{6}\end{align*}

**FIGURE 1.**
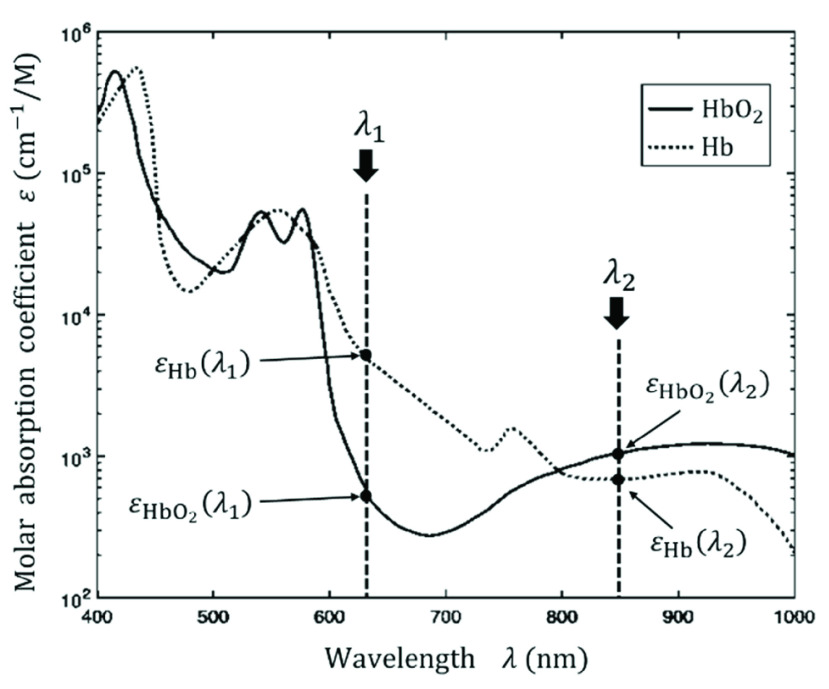
Relationship between molar absorption coefficient and light wavelength based on [16].

Here, the values of $\varepsilon _{\mathrm {Hb}\mathrm {O}_{2}}\left ({\lambda _{i} }\right) $ and $\varepsilon _{\mathrm {Hb}}\left ({\lambda _{i} }\right)$ are uniquely determined based on the relationship between the molar absorption coefficient and light wavelength, as shown in Fig. 1 [16]. Thus, estimating SpO_2_ from (6) is possible if $\mathrm {\Delta }A\left ({\lambda _{1} }\right)$ and $\Delta A\left ({\lambda _{2} }\right)$ are obtained.

**FIGURE 2.**
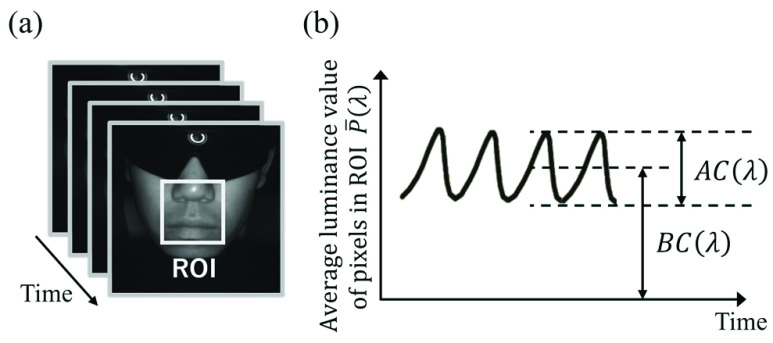
(a) Face image illuminated by light of wavelength $\lambda $ and (b) Video plethysmography (VPG).

Pulse waves related to heartbeat can be extracted from the temporal change in the luminance value of skin images, which is VPG. Fig. 2 shows an example of VPG. The VPG signal is obtained as the time variation of the average luminance $\bar {P}\left ({\lambda }\right)$ of all pixels included in the region of interest (ROI), which is a part of the face illuminated by light of a certain wavelength $\lambda $. Alternating $AC\left ({\lambda }\right)$ and bias $BC\left ({\lambda }\right)$ components of VPG are produced owing to the variation in hemoglobin levels in the blood and other factors, respectively. Here, the value of $AC\left ({\lambda }\right) $ normalized by $BC\left ({\lambda }\right)$ is defined by (7) and can be used as $\mathrm {\Delta }A\left ({\lambda }\right)$ in (6).

\begin{equation*} \Delta A\left ({\lambda }\right)=\frac {AC\left ({\lambda }\right)}{BC\left ({\lambda }\right)} \tag{7}\end{equation*}

Therefore, SpO_2_ can be measured contactlessly using (6) and (7) because $AC\left ({\lambda }\right)$ and $BC\left ({\lambda }\right)$ can be obtained from VPG.

To date, several studies have examined the non-contact measurement of SpO_2_ using skin images. Shao et al. [15] measured SpO_2_ contactlessly using the ratio of the reflected intensity of red and near-infrared light on a human face based on (6) and (7). Mishra et al. [17] separated a single light source into two components using a polarizing filter and estimated SpO_2_ using the ratio of the two components. Guazzi et al. [14] introduced skin-oxygen photoplethysmographic image analysis (Sophia), and used an automatic ROI selection algorithm. Wei et al. [18] applied blind source separation and independent component analysis of red–green–blue (RGB) signals from a camera and extracted the SpO_2_ signal with minimal noise. Kim et al. [19] attempted to estimate SpO_2_ by converting the color space of RGB signals to that of YCgCr. However, as mentioned, these methods need SpO_2_ calibration using a contact-type pulse oximeter to create an estimation model for each subject or subject group.

We conducted an experiment from a previous study presented in [15] to check the extent to which the estimation accuracy changed if reference values were not used. In the experiment, VPG signals (120 s) were obtained from three subjects, and then a linear regression model was created to estimate SpO_2_ based on the SpO_2_ reference values measured in the first 30 s. Table 1 presents the root mean square error (RMSE) of SpO_2_ estimated with and without the regression model. The results confirmed that without the regression model, the estimation accuracy is significantly reduced. Therefore, a method must be developed to estimate SpO_2_ values without reference values because reference values cannot be obtained in a real-world situation.

**TABLE 1:** RMSE of SpO_2_

With regression model	Without regression model
1.47%	5.02%

### B. Proposed Methods

In this study, we proposed two approaches for the non-contact estimation of SpO_2_ without reference values. The principles of each approach are described as follows:

#### 1) Method 1: Suppression of the Non-Pulsating Component

Let us consider the average luminance value of the pixels within the face area’s ROI illuminated by light of wavelength $\lambda $. It is expressed as follows:

\begin{equation*} \bar {P}\left ({\lambda }\right)=k\int {I\left ({\lambda }\right)s\left ({\lambda }\right)} d\lambda \tag{8}\end{equation*}

where $k$ and $s\left ({\lambda }\right)$, respectively, are the gain constant and spectral response function of the camera. Here, we assume that $s\left ({\lambda }\right)$ is a delta function with a peak at the center of the wavelength band of red (R) or near-infrared (NIR) light, expressed as follows:

\begin{equation*} s\left ({\lambda }\right)=\delta \left ({\lambda -\lambda _{i} }\right) \quad \left ({i=\mathrm {R}, \mathrm {NIR} }\right) \tag{9}\end{equation*}

After substituting (2), (3), and (9) into (8), we obtain (10):

\begin{align*}&\hspace {-.5pc} \log {\bar {P}\left ({\lambda _{i} }\right)}=\log k+\log {I_{0}\left ({\lambda _{i} }\right)}-A_{\mathrm {m}}\left ({\lambda _{i} }\right)-A_{0}\left ({\lambda _{i} }\right) \\&\qquad\qquad-\,\left \{{\varepsilon _{\mathrm {Hb}\mathrm {O}_{2}}\left ({\lambda _{i} }\right)c_{\mathrm {Hb}\mathrm {O}_{2}}+\varepsilon _{\mathrm {Hb}}\left ({\lambda _{i} }\right)c_{\mathrm {Hb}} }\right \}l \tag{10}\end{align*}

Here, the terms $\log k$, $\log {I_{0}\left ({\lambda _{i} }\right)}$, and $-A_{\mathrm {m}}\left ({\lambda _{i} }\right)$ do not vary with time because they are unrelated to the heartbeat. Therefore, these components can be suppressed by a band-pass filter that passes a heart rate-related band. The filtered signal of $\log {\bar {P}\left ({\lambda _{i} }\right)}$ can be expressed as follows:

\begin{align*}&\hspace {-.5pc} \log {\bar {P}_{\mathrm {filt}}\left ({\lambda _{i} }\right)}=-A_{0}\left ({\lambda _{i} }\right) \\& \qquad\qquad\qquad\qquad{ {\displaystyle { -\,\left \{{\varepsilon _{\mathrm {Hb}\mathrm {O}_{2}}\left ({\lambda _{i} }\right)c_{\mathrm {Hb}\mathrm {O}_{2}}+\varepsilon _{\mathrm {Hb}}\left ({\lambda _{i} }\right)c_{\mathrm {Hb}} }\right \}l } }} \tag{11}\end{align*}

The change in the average luminance $\bar {P}\left ({\lambda _{i} }\right)$ is opposite to that of the pulsating component of the absorbance $A_{AC}\left ({\lambda _{i} }\right)$; hence, we obtain,

\begin{equation*} \log {\bar {P}_{\mathrm {filt}}\left ({\lambda _{i} }\right)}=-A_{AC}\left ({\lambda _{i} }\right) \tag{12}\end{equation*}

**FIGURE 3.**
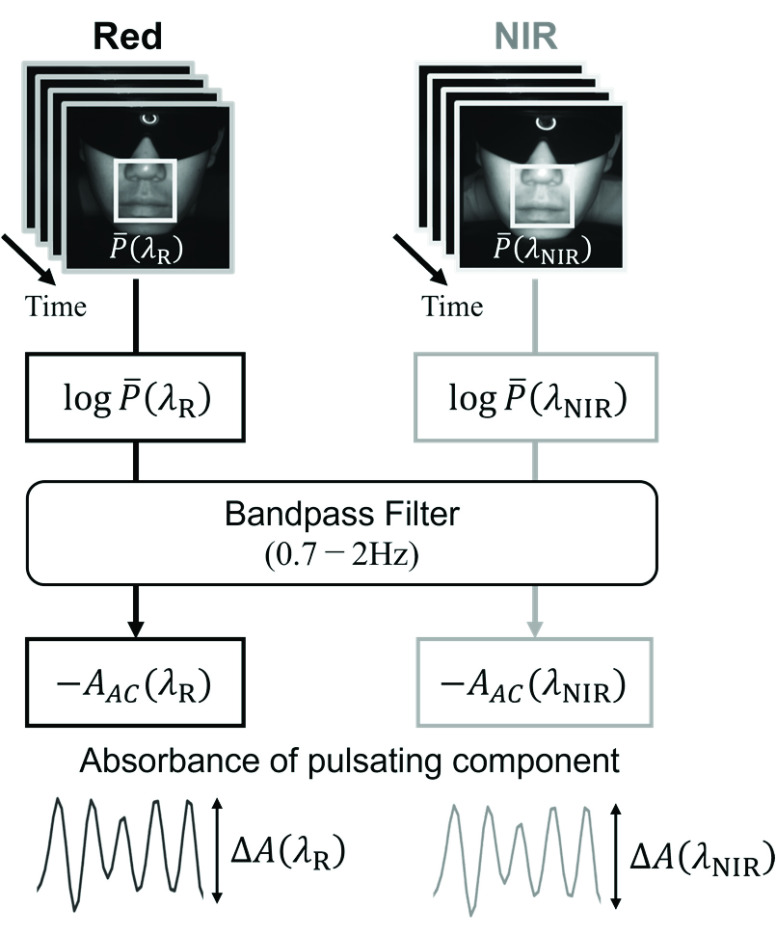
Calculation process of Method 1.

$\Delta A\left ({\lambda _{i} }\right)$ in (6) was obtained as the amplitude of $A_{AC}\left ({\lambda _{i} }\right)$; therefore, SpO_2_ can be calculated using (6). The process of Method 1 is illustrated in Fig. 3.

#### 2) Method 2: Utilizing the Difference in Light Penetration Depth

**FIGURE 4.**
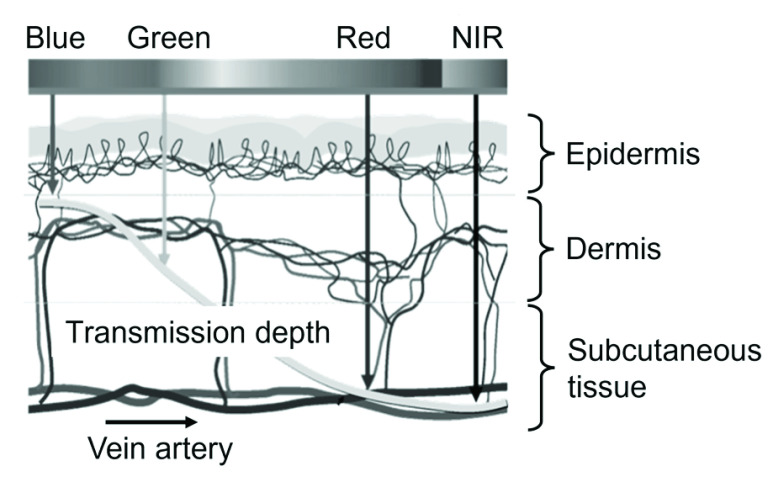
Wavelength-dependence on transmission depth in skin based on [21].

The depth of transmission when the skin is irradiated with light of different wavelengths is shown in Fig. 4 [21].

The pulsating component of the absorbance $A_{AC}\left ({\lambda _{\mathrm {G}} }\right)$ is given by (13) because green light is irradiated only up to the middle of the dermis layer, as shown in Fig. 4. Conversely, red and NIR light penetrate the subcutaneous tissue with its arteries and veins. Thus, (14) estimates the pulsating components of the red and NIR light absorbance.

\begin{align*} A_{AC}\left ({\lambda _{\mathrm {G}} }\right)&=A_{0}\left ({\lambda _{\mathrm {G}} }\right) \tag{13}\\ A_{AC}\left ({\lambda _{i} }\right)&=A_{0}\left ({\lambda _{i} }\right)+\left \{{\varepsilon _{\mathrm {Hb}\mathrm {O}_{2}}\left ({\lambda _{i} }\right)c_{\mathrm {Hb}\mathrm {O}_{2}}+\varepsilon _{\mathrm {Hb}}\left ({\lambda _{i} }\right)c_{\mathrm {Hb}} }\right \}l \\ &\qquad \qquad \qquad \qquad \qquad (i=\mathrm {R}, \mathrm {NIR}) \tag{14}\end{align*}

Here, we attempted to separate the pulsating components of the absorbance in the shallow layer from those in the deep layer using PCA. We focused on the fact that the absorbance in the shallow layers, such as the epidermis and dermis, exists as a common term, i.e., $A_{0}(\lambda _{i})$ for all three light wavelengths. In this study, we propose two methods (i.e., 2A and 2B) based on the differences in applying PCA.

**FIGURE 5.**
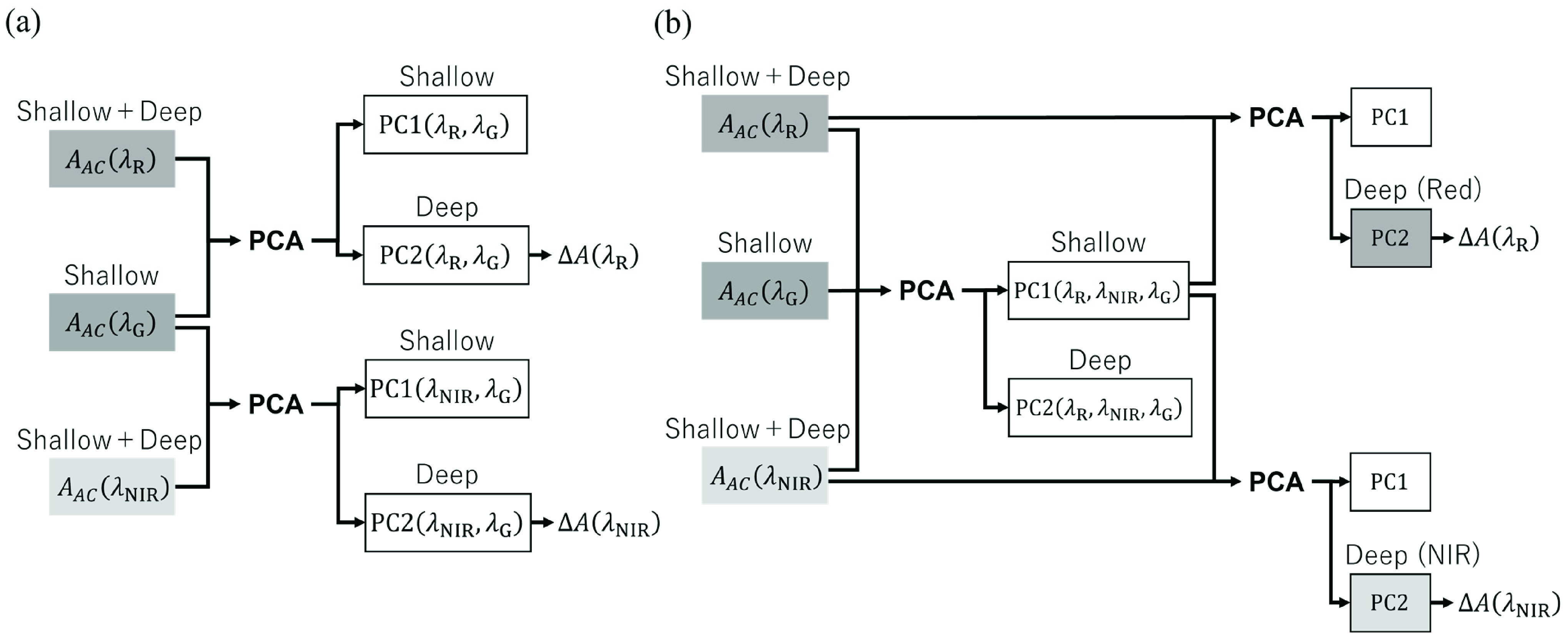
Calculation processes of (a) Method 2A and (b) Method 2B.

Fig. 5(a) shows the calculation process for Method 2A. We considered that when PCA was performed for $A_{AC}\left ({\lambda _{\mathrm {R}} }\right)$ and $A_{AC}\left ({\lambda _{\mathrm {G}} }\right)$, the information in the shallow layer, which was common to all light wavelengths, was obtained as the first principal component $\mathrm {PC1}\left ({\lambda _{\mathrm {R}}, \lambda _{\mathrm {G}} }\right);$ meanwhile, the information in the deep layer, which was obtained only from the red light, was obtained as the second principal component $\mathrm {PC2}\left ({\lambda _{\mathrm {R}}, \lambda _{\mathrm {G}} }\right)$. The same operations were performed for $A_{AC}\left ({\lambda _{\mathrm {NIR}} }\right)$ and $A_{AC}\left ({\lambda _{\mathrm {G}} }\right)$. The changes in the absorbance of arterial blood $\mathrm {\Delta }A\left ({\lambda _{\mathrm {R}} }\right)$ and $\mathrm {\Delta }A\left ({\lambda _{\mathrm {NIR}} }\right)$ were calculated by taking the amplitudes of $\mathrm {PC2}\left ({\lambda _{\mathrm {R}}, \lambda _{\mathrm {G}} }\right)$ and $\mathrm {PC2}\left ({\lambda _{\mathrm {NIR}}, \lambda _{\mathrm {G}} }\right)$, respectively, and SpO_2_ was estimated using these components.

Fig. 5(b) shows the calculation procedure for Method 2B. First, PCA was applied to all three signals, i.e., $A_{AC}\left ({\lambda _{\mathrm {R}} }\right)$, $A_{AC}\left ({\lambda _{\mathrm {NIR}} }\right)$, and $A_{AC}\left ({\lambda _{\mathrm {G}} }\right)$, and shallow layer information was extracted as the first principal component $\mathrm {PC1}\left ({\lambda _{\mathrm {R}}, \lambda _{\mathrm {NIR}}, \lambda _{\mathrm {G}} }\right)$. Second, the information in the deep layer was extracted as the second principal component PC2 when PCA was applied to $A_{AC}\left ({\lambda _{\mathrm {R}} }\right)$ and $\mathrm {PC1}\left ({\lambda _{\mathrm {R}}, \lambda _{\mathrm {NIR}}, \lambda _{\mathrm {G}} }\right)$, and applied to $A_{AC}\left ({\lambda _{\mathrm {NIR}} }\right)$ and $\mathrm {PC1}\left ({\lambda _{\mathrm {R}}, \lambda _{\mathrm {NIR}}, \lambda _{\mathrm {G}} }\right)$. Finally, SpO_2_ was estimated as described in Method 2A.

## III. Experiment

### A. Evaluation Indicators

In this study, the RMSE and correlation coefficient (CC) between the reference and estimated values of SpO_2_ were used to evaluate the estimation accuracy of the proposed methods. The essential performance of a pulse oximeter, as defined in ISO 80601-2-61 [22], is that the RMSE of 66.6% of all data should be within 4%. In addition, the 4C mortality score constructed by Knight et al. [23], which predicts mortality risk in COVID-19 patients, indicates that an SpO_2_ value less than 92% is abnormal. Based on these two criteria, we set the target value of the RMSE in the present study to be within 4%.

**FIGURE 6.**
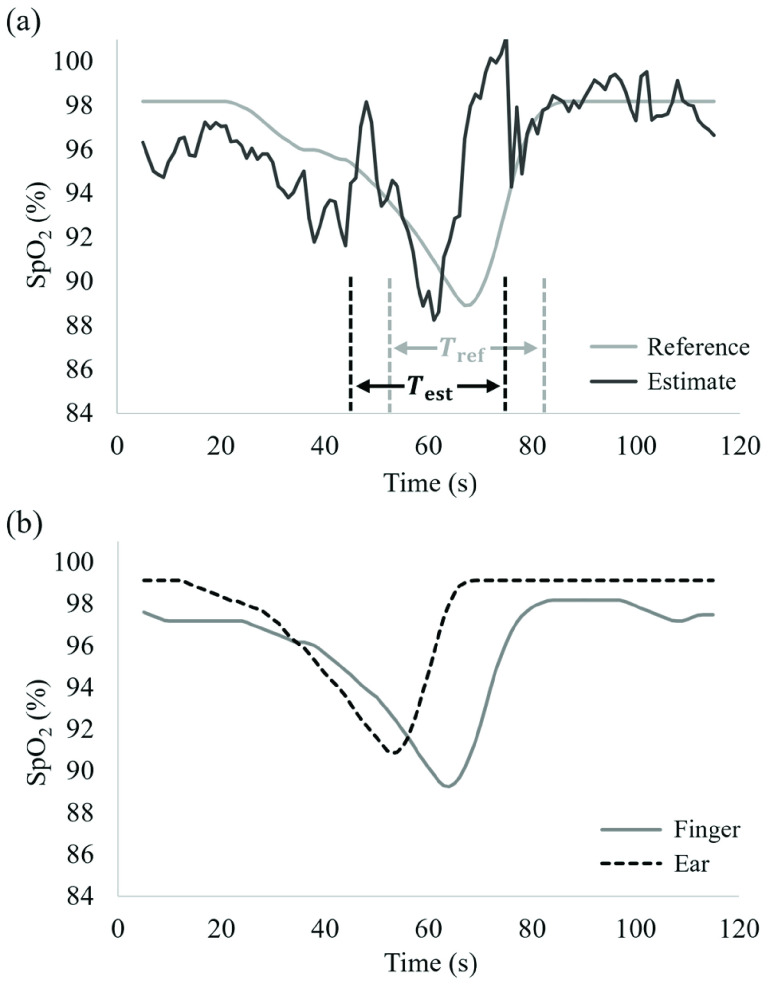
(a) Comparison between reference (fingertip) and estimated values of SpO_2_ and (b) comparison between SpO_2_ values measured with contact-type oximeters attached at fingertip and ear.

Fig. 6(a) shows examples of the reference and estimated values of SpO_2_. The conventional method described in [15] was used for estimation. In Fig. 6(a), the time lag between the reference and estimated values is shown. We hypothesized that this is due to the difference in the measurement site between the reference and estimated values. To check this hypothesis, we measured SpO_2_ values simultaneously using finger and ear clip pulse oximeters, which are contact-type sensors for clinical use. Fig. 6(b) shows the comparison of SpO_2_ values obtained from the finger and ear. As can be seen, the time lag in SpO_2_ value is observed at different measurement sites even if contact-type sensors were used.

From these results, we considered that it was necessary to evaluate the estimation accuracy after shifting the SpO_2_ signals to eliminate the time lag between the reference and estimated values. As shown in Fig. 6 (a), we respectively defined $T_{\mathrm {ref}}$ and $T_{\mathrm {est}}$ as the periods (15 s) before and after the minimum points of the reference and estimated values. Then, we calculated the RMSE and CC for the data included in the $T_{\mathrm {ref}}$ and $T_{\mathrm {est}}$. periods.

### B. Experimental Setup

**FIGURE 7.**
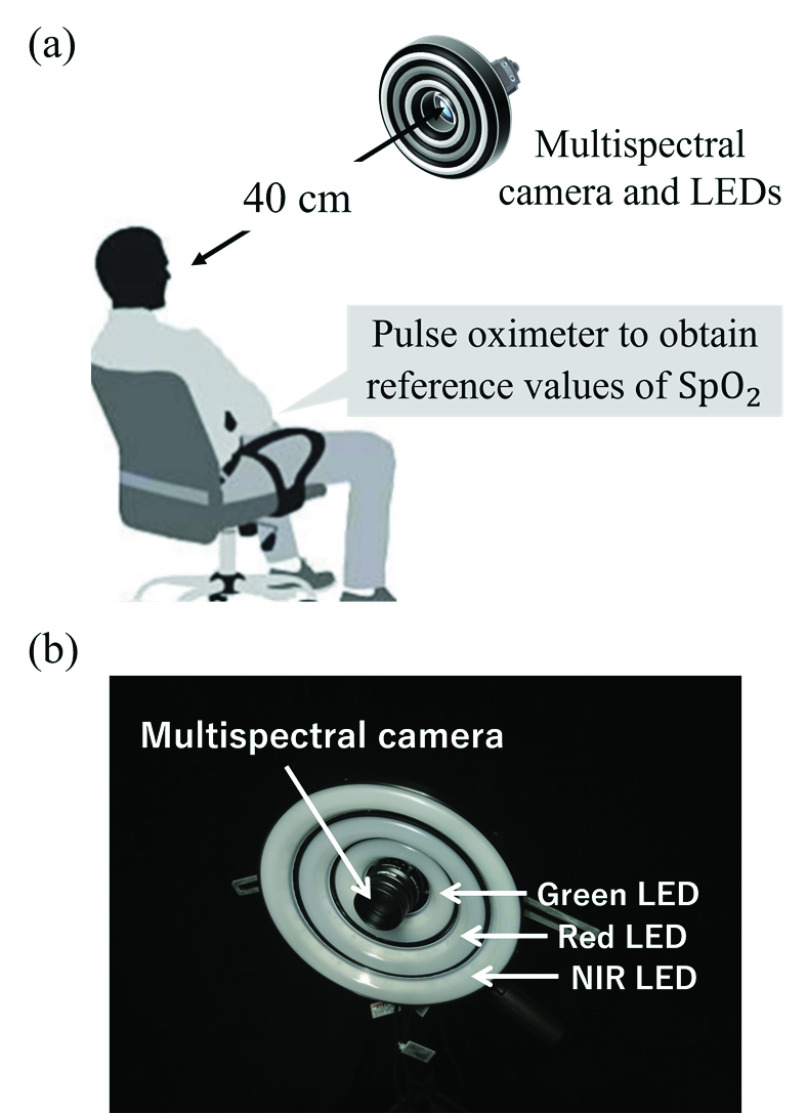
(a) Experimental setup and (b) multispectral camera with three ring-shaped LEDs.

The experimental setup is illustrated in Fig. 7(a). In the resting state, a subject’s face was illuminated by three ring-shaped light-emitting diodes (LEDs) as shown in Fig. 7(b) and photographed with a multispectral camera (msCAM snapshot RGB-NIR model, Spectral DEVICES Inc.) [24]. The wavelength centers of the LED light were 524 nm (green), 630 nm (red), and 850 nm (NIR). There were no light sources other than the LEDs in the darkened room. The camera settings are listed in Table 2.

**TABLE 2:** Settings of Multispectral Camera

Settings	Values
Centers of light wavelengths	450 nm, 550 nm, 650 nm, 800 nm
Number of pixels	$512\times512$ pixels/band
Frame rate	10 fps
Exposure time	100 ms
Bit depth	8-bit/band
Saving format	avi (uncompressed)

There were 14 male subjects and 1 female, with an average age of 23.7±1.0 years. A finger-clip sensor was attached to one of each subject’s fingertips to measure the reference value of SpO_2_ (ML/320F, ADInstruments). The sampling rate of the reference SpO_2_ was 1 Hz. The face video image and reference SpO_2_ signal were measured three times for each subject, and the duration of each measurement was 120 s. The subjects were instructed to cease breathing during the measurement to decrease their SpO_2_ level intentionally. A 10 s window was applied to the data and shifted by 1 s so that the value of SpO_2_ was estimated every second. The values of the molar absorption coefficient, $\varepsilon _{\mathrm {Hb}\mathrm {O}_{2}}\left ({\lambda }\right) $ and $\varepsilon _{\mathrm {Hb}}\left ({\lambda }\right)$, used to calculate SpO_2_ are shown in Table 3.

**TABLE 3:** Values of Molar Absorption Coefficient

	$\lambda = 630 \mathrm {nm}$ (red)	$\lambda = 850 \mathrm {nm}$ (NIR) (red)
HbO_2_	$610.0\,\,\mathrm {L}\cdot {\mathrm {mol}}^{-1}\cdot {\mathrm {cm}}^{-1}$	$1058.0\,\,\mathrm {L}\cdot {\mathrm {mol}}^{-1}\cdot {\mathrm {cm}}^{-1}$
Hb	$5148.8\,\,\mathrm {L}\cdot {\mathrm {mol}}^{-1}\cdot {\mathrm {cm}}^{-1}$	$691.3\,\,\mathrm {L}\cdot {\mathrm {mol}}^{-1}\cdot {\mathrm {cm}}^{-1}$

In addition, we conducted another experiment in a room with ambient light to verify the estimation accuracy in a real-world environment. The illuminance on the subject’s face was 111 lux. Except for the ambient light, the setting was identical to the previous experiment. The subjects of this experiment were one female and 19 males aged 23.6 ± 1.0 years.

All the subjects who participated in the two experiments had medium tan skin and no respiratory diseases. All provided informed written consent and the experiment was approved by the Internal Review Board of Tohoku University, Japan (ID: 22A-13).

## IV. Results

**FIGURE 8.**
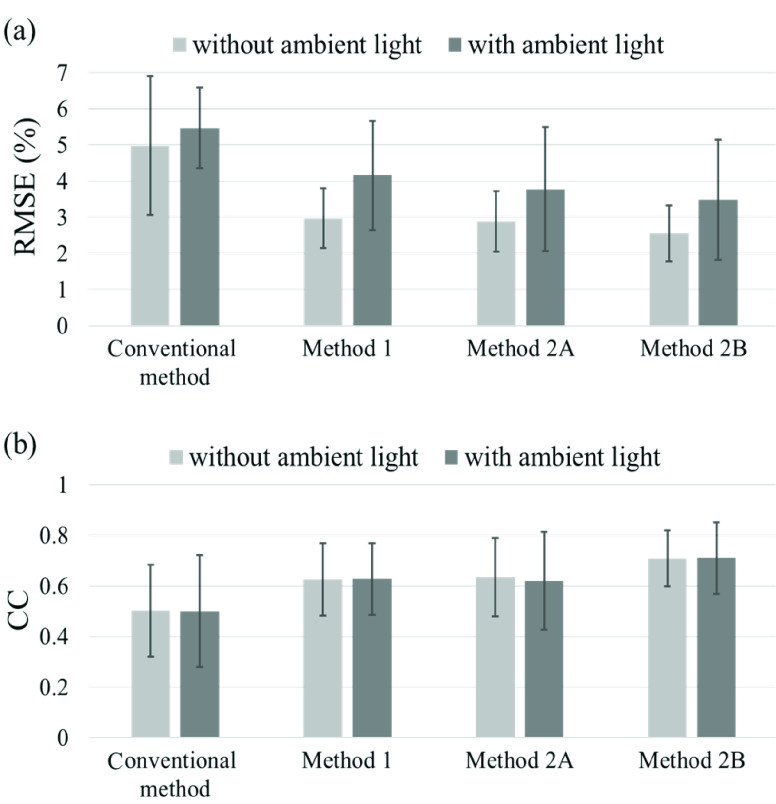
Comparison of (a) root mean square error (RMSE) and (b) correlation coefficient (CC) between methods with and without ambient light.

The RMSEs between the methods (conventional method [15], Method 1, Method 2A, and Method 2B) and that of CC are compared and shown in Fig. 8. This is the result obtained without any correction by reference values.

From Fig. 8(a), we can see that the RMSEs of all the proposed methods are lower than those of the conventional method. Under ideal dark-room conditions, the RMSE of Method 2B was the lowest (2.55%). Although the RMSE of the proposed methods increased by approximately 1% and a standard deviation of the RMSE, which means individual differences increased if the data were measured with ambient light, the RMSEs of the proposed Methods 2A and 2B were within 4%. Moreover, Fig. 8(b) shows that the CCs of the proposed methods were higher than those of the conventional method. This also indicates that the CC is less affected by ambient light.

**FIGURE 9.**
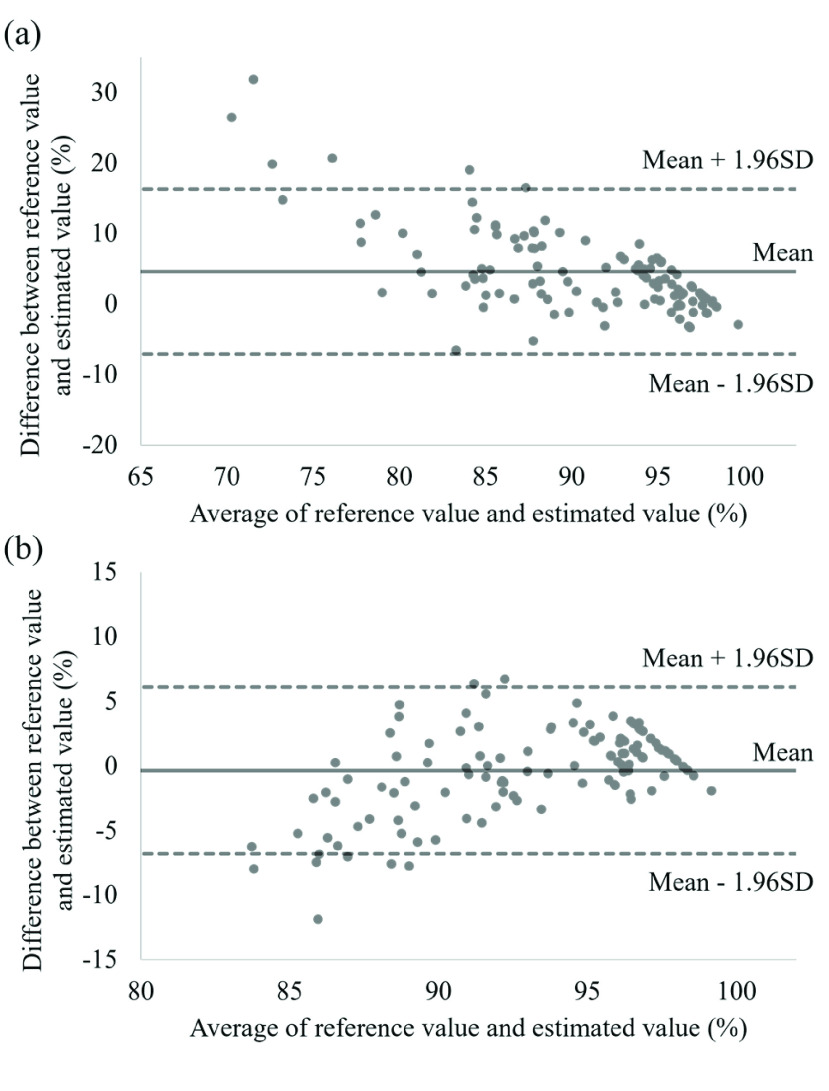
Bland–Altman plots of (a) conventional method and (b) Method 2B with ambient light.

The Bland–Altman plots of the conventional method and Method 2B are shown in Fig. 9. The plot data were obtained in a room with ambient light. For both methods, Bland–Altman plots have limits of agreement of 95% representing the agreement between the reference and the estimated values of SpO_2_. Conversely, we can confirm that compared to the conventional method, the systematic error is suppressed by the proposed method.

## V. Discussion

In the conventional method, the approximation shown in (15), which is the Maclaurin series expansion, was used to estimate SpO_2_.

\begin{equation*} -\log {\left ({1-\frac {\Delta I\left ({\lambda }\right)}{I\left ({\lambda }\right)} }\right)\cong \frac {\Delta I\left ({\lambda }\right)}{I\left ({\lambda }\right)}} \tag{15}\end{equation*}

where $I\left ({\lambda }\right) $ and $\mathrm {\Delta }I\left ({\lambda }\right)$ are the intensity and changing range of the reflected light intensity, respectively, when the skin is irradiated with light of wavelength $\lambda $. The approximation in (15) is valid when $I\left ({\lambda }\right)$ is sufficiently large compared to $\mathrm {\Delta }I\left ({\lambda }\right)$. $I\left ({\lambda }\right)$ and $\Delta I\left ({\lambda }\right)$ correspond to the non-pulsating and pulsating components, respectively, which are the $BC$ and $AC$ of VPG. Therefore, when the $BC$ reduces owing to low incident light intensity or shadows on the face, the estimation accuracy deteriorates because the approximation error increases.

Furthermore, we examined why Method 1 improved the estimation accuracy compared to the conventional method. Method 1 attempts to suppress non-pulsating components ( $BC$) by applying a band-pass filter to the natural logarithm of the averaged pixel luminance value. This is to extract only the heartbeat-related component that reflects changes in the amount of hemoglobin in the blood vessel. This approach is considered to suppress the effects of fluctuating incident light and shadows on the face, which are not related to the amount of hemoglobin.

The influence of incident light and the absorbance of melanin are eliminated by applying a band-pass filter based on the assumption that they do not change with time. However, if the subject’s face moves during the measurement, these influences are possibly not completely eliminated. Otherwise expressed, the noise caused by moving the ROI could not be completely removed. In addition, the pulsation component in the shallow layer, caused by capillaries in the dermis layer, cannot be removed using Method 1.

In contrast, in Method 2, by applying PCA, we attempted to extract a common component included in VPG with different light wavelengths. This operation is considered to improve accuracy because noise, such as the pulsation component in the shallow layer and the effect of facial motion, were separated as the first principal components. These components are difficult to remove by using a simple band-pass filter because their influences on $c_{\mathrm {Hb}}$ and $c_{\mathrm {Hb}\mathrm {O}_{2}}$ may differ. Actually, except for one subject, the RMSE of Method 2 was lower than that of Method 1, and this did not depend on the difference of lighting conditions. This means that Method 2 performs better in estimating SpO_2_ than Method 1 regardless of subjects or light conditions. On the contrary, the calculation of Method 1 is simpler than Method 2 because it does not use PCA; therefore, Method 1 is easy to implement in embedded systems.

This study had certain limitations. First, all the participants had the same skin color (medium tan), and no investigation was done to determine the effect of differences in skin color on the estimation accuracy. It has been reported that the difference in skin colors affects SpO_2_ values measured with a contact-type pulse oximeter [25]. To counter this, either more data must be collected from subjects with different skin tones and/or a correction formula of SpO_2_ values based on a skin color classification model such as Fitzpatrick skin typing is needed [26]. Furthermore, the U.S. Food and Drug Administration has reported that poor circulation, skin thickness, skin temperature, and present tobacco use can affect the accuracy of a pulse oximeter [27]; therefore, we should consider these factors when using the proposed method.

Second, the estimation accuracy in a real-world environment must be improved. The RMSE increased by approximately 1% under normal ambient light. This result indicates that light irradiation from the outside has a strong impact on estimation accuracy. Thus, a method must be developed that can accurately estimate SpO_2_, even under conditions with several lighting sources, such as incandescent lamps or sunlight.

Finally, we should investigate the effect of differences in measurement points on SpO_2_. In this study, we found that there was a time lag in the change of SpO_2_ between different body parts, i.e., the finger and ear. In the case of applying Method 2B to data with ambient light, the RMSE without lag correction was 3.2%, which was 0.7% lower than with lag correction. To judge the validity of this correction, the reference values of SpO_2_ measured at the nose and mouth must be confirmed because these areas were included in the ROI to obtain the VPG. In addition, we should assess the estimation accuracy when the proposed method is applied daily to people with low SpO_2_ e.g., patients with COPD, because intentionally holding the breath during an experiment may affect the estimation accuracy.

If a remote oximeter system can be realized by the proposed method, screening for potential infections in people would be similar to checking body temperatures at the entrances to public facilities during the Covid-19 pandemic. In addition, the proposed system will be useful to detect the obstructive sleep apnea syndrome (OSAP) [28], [29] at an early stage if the face of a sleeping person can be captured. This is effective to reduce cardiovascular and cerebrovascular risks related to the OSAP. Moreover, remote oximeters could be used to monitor the vital signs of patients with chronic respiratory diseases. For example, if the blood oxygen levels of the patients could be monitored daily with the remote oximeter incorporated into a washstand mirror, it should be possible to detect worsening symptoms. Technologies to capture the state of the human body without wearing sensors have great potentials for early detection of diseases that do not show subjective symptoms at an early stage. This will not only improve the quality of life of patients, but also contribute to the future development of preventive medicines.

Furthermore, e-health and m-health [30] using general-purpose communication devices such as smartphones have been rapidly expanding in recent years. Technologies to acquire high-quality biometric information using a general-purpose camera allow telemedicine physicians to make diagnoses based on objective biometric information in addition to conventional medical interviews. This kind of technique can make a significant contribution to improving the quality of telemedicine.

## VI. Conclusion

In this study, we proposed new methods for non-contact SpO_2_ measurement that do not require reference values and conducted experiments to evaluate their estimation accuracy. The experimental results showed that the proposed methods could estimate SpO_2_ with a lower RMSE and a higher CC compared to those of the conventional method. In addition, the RMSEs of the proposed methods were within 4% even in a room with normal ambient light.
